# Effects of simvastatin on iNOS and caspase-3 levels and oxidative stress following smoke inhalation injury

**DOI:** 10.3892/mmr.2020.11621

**Published:** 2020-10-21

**Authors:** Rong-Qiang Yang, Peng-Fei Guo, Zhao Ma, Cheng Chang, Qing-Nan Meng, Ya Gao, Imran Khan, Xiao-Bo Wang, Zheng-Jun Cui

Mol Med Rep 22: 3405-3417, 2020; DOI: 10.3892/mmr.2020.11413

After the publication of the above paper, the authors have noticed that the affiliations were presented incorrectly; essentially, Drs Rong-qiang Yang, Peng-fei Guo, Qing-nan Meng, Ya Gao, Imran Khan, Xiao-bo Wang and Zheng-jun Cui are based at the Department of Burn and Repair Reconstruction Surgery, The First Affiliated Hospital of Zhengzhou University, whereas Drs Zhao Ma and Cheng Chang are located at The School of Basic Medical Science of Zhengzhou University. Therefore, the affiliations for this paper should have appeared as follows:

RONG-QIANG YANG^1^, PENG-FEI GUO^1^, ZHAO MA^2^, CHENG CHANG^2^, QING-NAN MENG^1^, YA GAO^1^, IMRAN KHAN^1^, XIAO-BO WANG^1^ and ZHENG-JUN CUI^1^

^1^Department of Burn and Repair Reconstruction Surgery, The First Affiliated Hospital of Zhengzhou University; ^2^The School of Basic Medical Science of Zhengzhou University, Zhengzhou, Henan 450052, P.R. China

The authors regret that these errors with the author affiliations were not noticed prior to the publication of their paper, and apologize for any inconvenience caused.

